# Supplementary material for: The GET insertase exhibits conformational plasticity and induces membrane thinning
Source: Nat Commun. 2023 Nov 14;14:7355. doi: 10.1038/s41467-023-42867-2 (PMC10646013; doi:10.1038/s41467-023-42867-2)
Supplement: Supplementary file 1 — Supplementary Information [file 41467_2023_42867_MOESM1_ESM.pdf]

Supplementary Figures

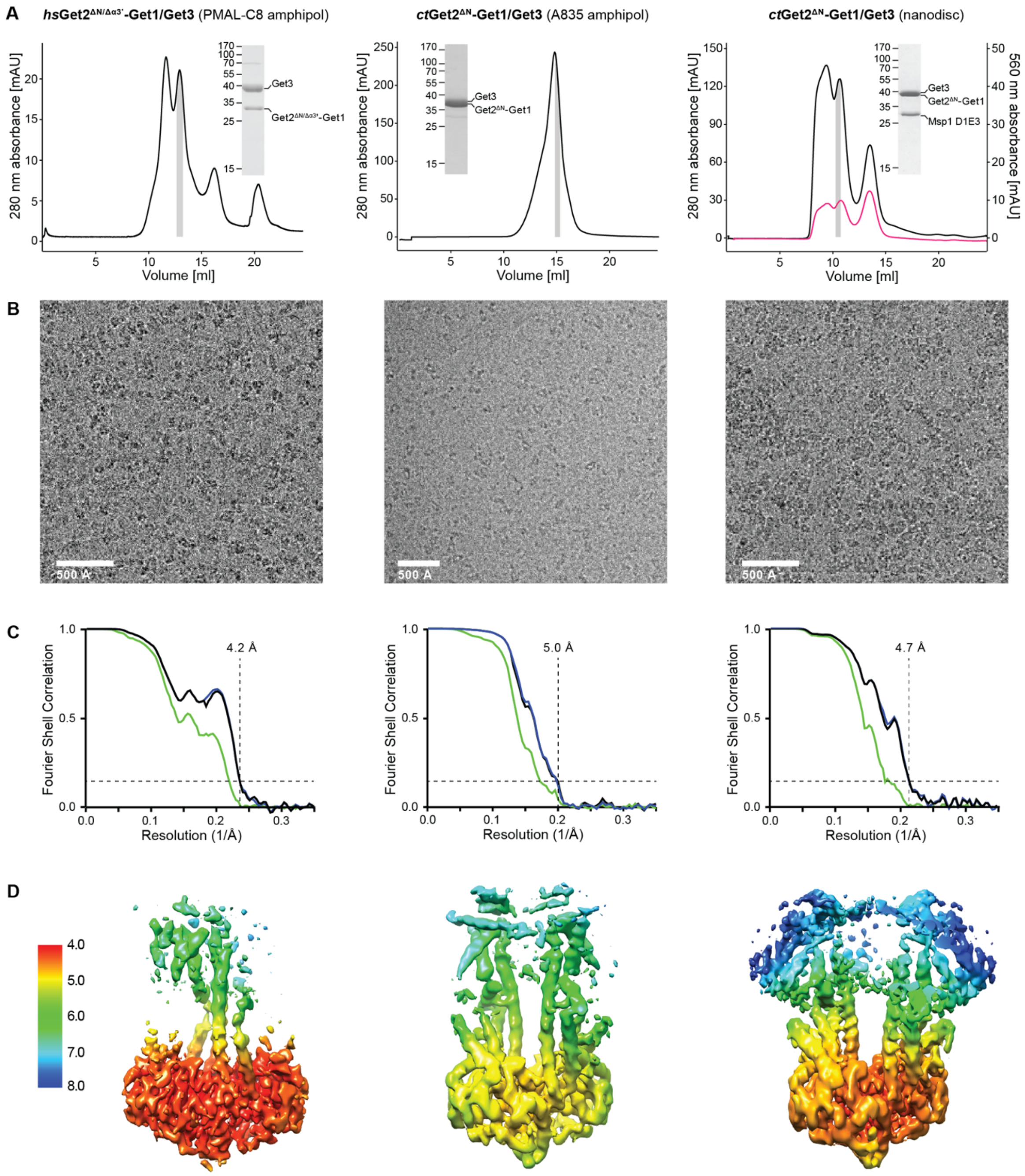

<u>Supplementary Figure 1</u> – Cryo-EM sample preparation and experimental quality.

A) Size exclusion chromatography profiles for the indicated complexes. The single fraction taken for cryo-EM is indicated in grey. The insets show Coomassie stained 12.5% SDS-PAGE gels for the final samples. **B)** Representative micrographs for each complex. **C)** Gold-standard FSC curves for the final reconstruction. For each reconstruction, the no mask (green), tight mask (blue) and corrected (black) curves are shown. **D)** Local resolution estimates of the final reconstructions for each complex.

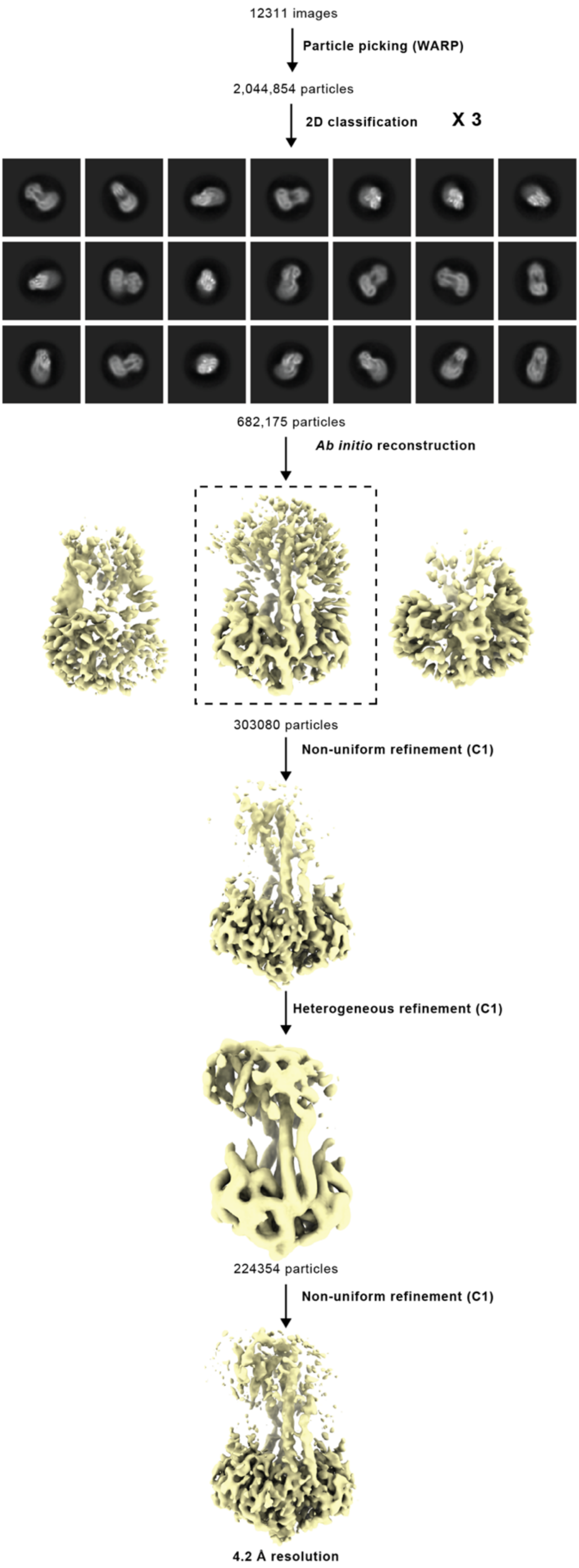
12311 images
Particle picking (WARP)
2,044,854 particles
2D classification
X 3
682,175 particles
Ab initio reconstruction
303080 particles
Non-uniform refinement (C1)
Heterogeneous refinement (C1)
224354 particles
Non-uniform refinement (C1)
4.2 Å resolution

<u>Supplementary Figure 2</u> – Workflow for single-particle cryo-EM data processing for *hs*Get2$^{\Delta N/\Delta\alpha 3'}$-Get1/Get3 in PMAL-C8.

Particles were autopicked using WARP, then subjected to 2D classification in cryoSPARC. An initial 3D reconstruction generated *ab initio* was subjected to heterogeneous refinement and non-uniform refinement, resulting in a reconstruction with an average resolution of 4.2 Å.

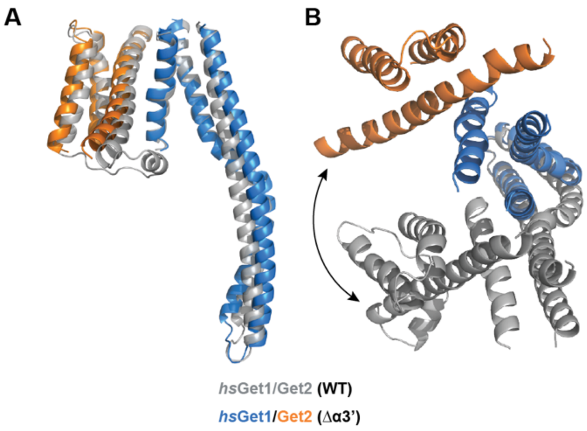

<u>Supplementary Figure 3</u> – Structural features of *hs*Get2$^{\Delta N/\Delta\alpha 3'}$-Get1/Get3.

A) Superimposition of the wild type (WT) and Δα3' *hs*Get1/Get2 heterodimer structures (RMSD 2.93 Å over 214 Cα atoms) shows a conserved fold. **B)** View of equivalent WT and Δα3' *hs*Get1/Get2 heterodimers from the luminal side of the membrane after superimposition via *hs*Get3 (RMSD 1.71 Å over 503 Cα atoms). The arrow highlights the relative positional variation between the two heterodimers.

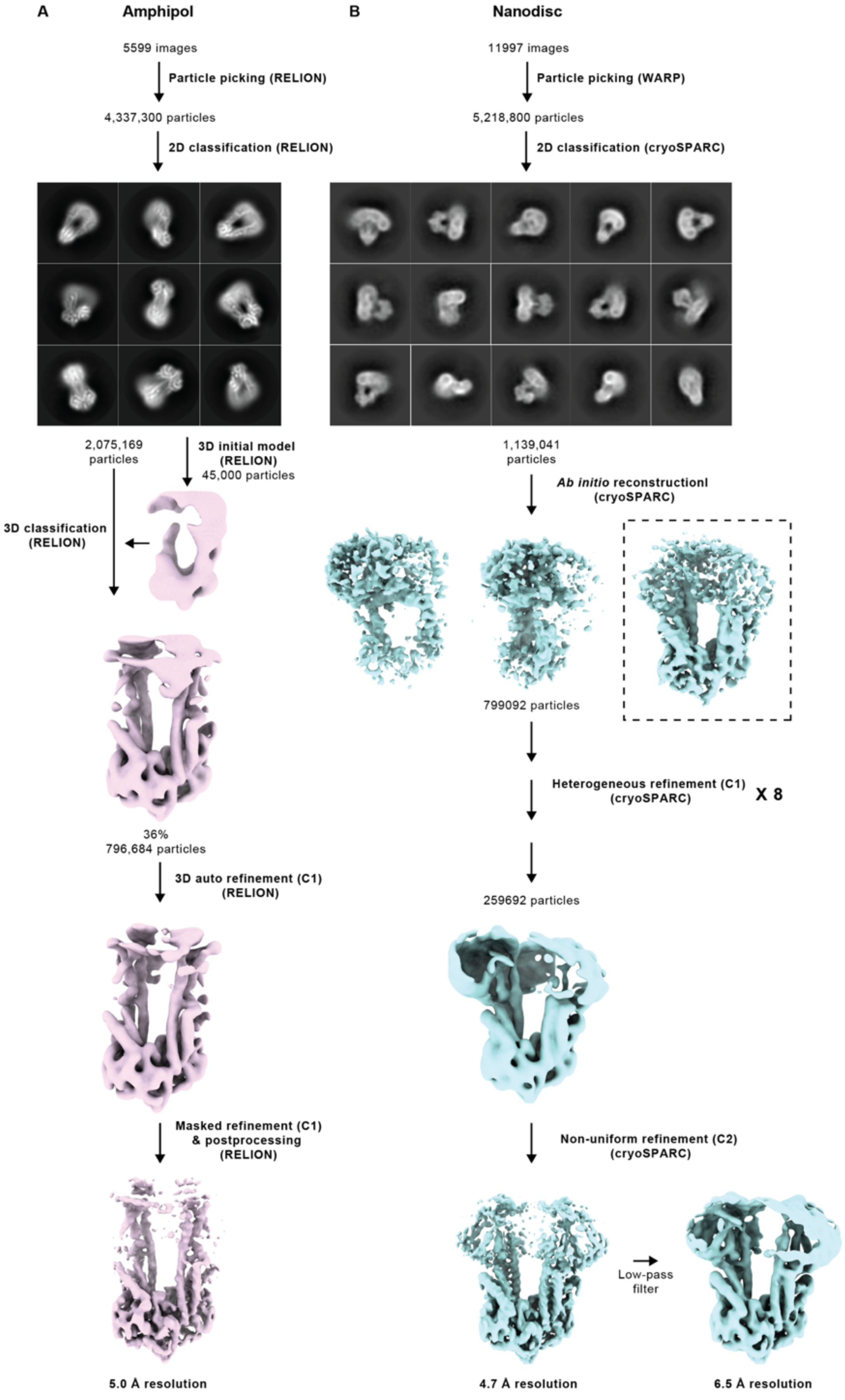

A
Amphipol
5599 images
Particle picking (RELION)
4,337,300 particles
2D classification (RELION)
2,075,169 particles
3D initial model (RELION)
45,000 particles
3D classification (RELION)
36%
796,684 particles
3D auto refinement (C1) (RELION)
Masked refinement (C1) & postprocessing (RELION)
5.0 Å resolution
B
Nanodisc
11997 images
Particle picking (WARP)
5,218,800 particles
2D classification (cryoSPARC)
1,139,041 particles
Ab initio reconstructionl (cryoSPARC)
799092 particles
Heterogeneous refinement (C1) (cryoSPARC)
X 8
259692 particles
Non-uniform refinement (C2) (cryoSPARC)
Low-pass filter
4.7 Å resolution
6.5 Å resolution

<u>Supplementary Figure 4</u> – Workflow for single-particle cryo-EM data processing for *ct*Get2$^{\Delta N}$-Get1/Get3 complexes.

A) For the amphipol complex, particles were autopicked in Relion3.0, then subjected to 2D classification followed by 3D classification against a low-resolution *de novo* model. Particle images were then refined using a soft mask, resulting in a reconstruction with an average resolution of 5.0 Å. **B)** For the nanodisc complex, particles were autopicked in WARP, then subjected to 2D classification in cryoSPARC. An initial 3D reconstruction generated *ab initio* was subjected to multiple rounds of heterogeneous refinement followed by non-uniform refinement, resulting in a reconstruction with an average resolution of 4.7 Å. More complete density for low resolution regions was present in a reconstruction low-pass filtered to 6.5 Å.

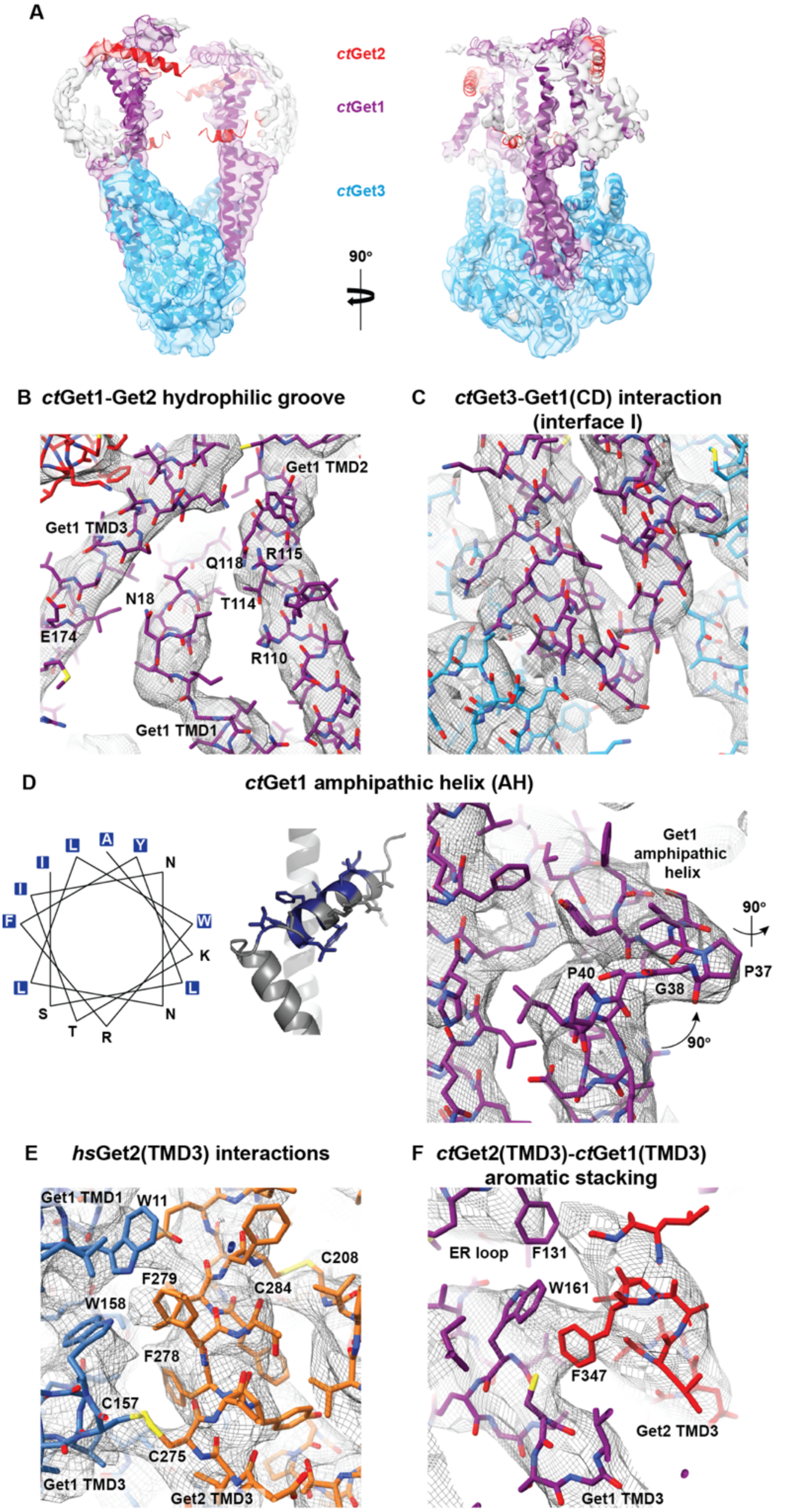
A
ctGet2
ctGet1
ctGet3
90°
B ctGet1-Get2 hydrophilic groove
Get1 TMD2
Get1 TMD3
Q118
R115
N18
T114
E174
R110
Get1 TMD1
C ctGet3-Get1(CD) interaction (interface I)
D
ctGet1 amphipathic helix (AH)
L
A
Y
I
N
I
F
W
K
L
L
S
T
R
N
Get1 amphipathic helix
90°
P40
G38
P37
90°
E hsGet2(TMD3) interactions
Get1 TMD1
W11
C208
F279
C284
W158
F278
C157
C275
Get1 TMD3
Get2 TMD3
F ctGet2(TMD3)-ctGet1(TMD3) aromatic stacking
ER loop
F131
W161
F347
Get2 TMD3
Get1 TMD3

Supplementary Figure 5 – Structural features of the *ct*Get2$^{\Delta N}$-Get1/Get3 and *hs*Get2$^{\Delta N}$-Get1/Get3 complexes.

A) Model for *ct*Get2$^{\Delta N}$-Get1/Get3 in nanodiscs superposed to the cryo-EM density. **B-C)** Representative density for the **B)** hydrophilic groove and **C)** *ct*Get1-CD interface with *ct*Get3 in the amphipol *ct*Get2$^{\Delta N}$-Get1/Get3 reconstruction **D)** The amphipathic helix of *ct*Get1 (residues 22-36) shown in both helical wheel and structural representations. Hydrophobic residues are coloured blue. **E-F)** Representative density for **E)** *hs*Get2$^{\Delta N}$-Get1/Get3 and **F)** amphipol *ct*Get2$^{\Delta N}$-Get1/Get3 showing the region around the absolutely conserved tryptophan of Get1 TMD3.

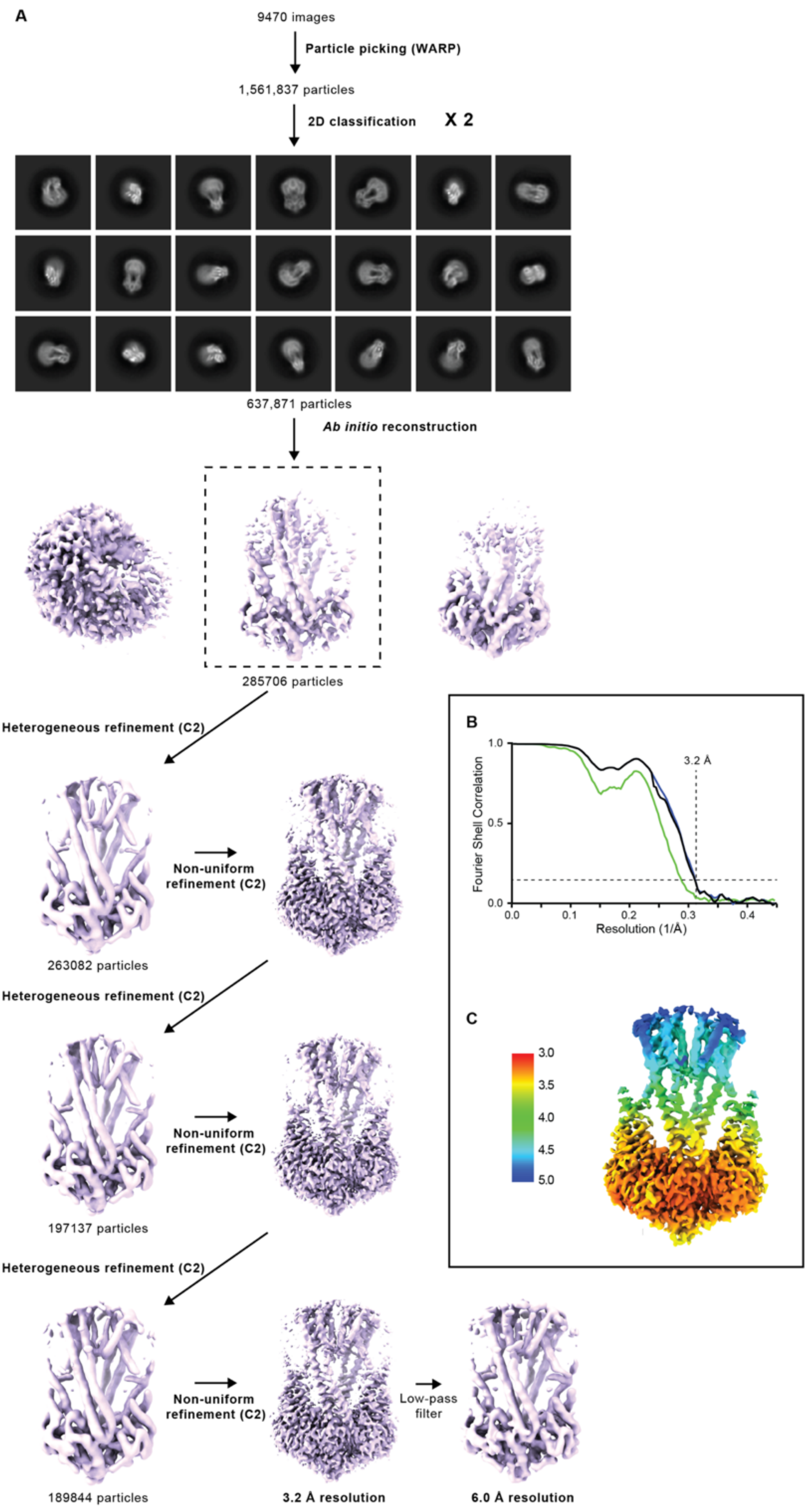
A
9470 images
Particle picking (WARP)
1,561,837 particles
2D classification
X 2
637,871 particles
Ab initio reconstruction
285706 particles
Heterogeneous refinement (C2)
Non-uniform refinement (C2)
263082 particles
Heterogeneous refinement (C2)
Non-uniform refinement (C2)
197137 particles
Heterogeneous refinement (C2)
Non-uniform refinement (C2)
Low-pass filter
189844 particles
3.2 Å resolution
6.0 Å resolution
B
Fourier Shell Correlation
Resolution (1/Å)
3.2 Å
C
3.0
3.5
4.0
4.5
5.0

<u>Supplementary Figure 6</u>–Improved resolution of the *hs*Get2$^{\Delta N}$-Get1/Get3 reconstruction. **A)** Pipeline for re-processing of the single-particle cryo-EM dataset (EMDB-10266)[24]. Particles were autopicked using WARP, then subjected to 2D classification in cryoSPARC. An initial 3D reconstruction generated *ab initio* was subjected to multiple rounds of heterogeneous refinement and non-uniform refinement, resulting in a reconstruction with an average resolution of 3.2 Å. More complete density for low resolution regions was present in a reconstruction low-pass filtered to 6.0 Å. **B)** Gold-standard FSC curves for the final reconstruction, showing the no mask (green), tight mask (blue) and corrected (black) curves. **C)** Local resolution estimate for the final reconstruction.

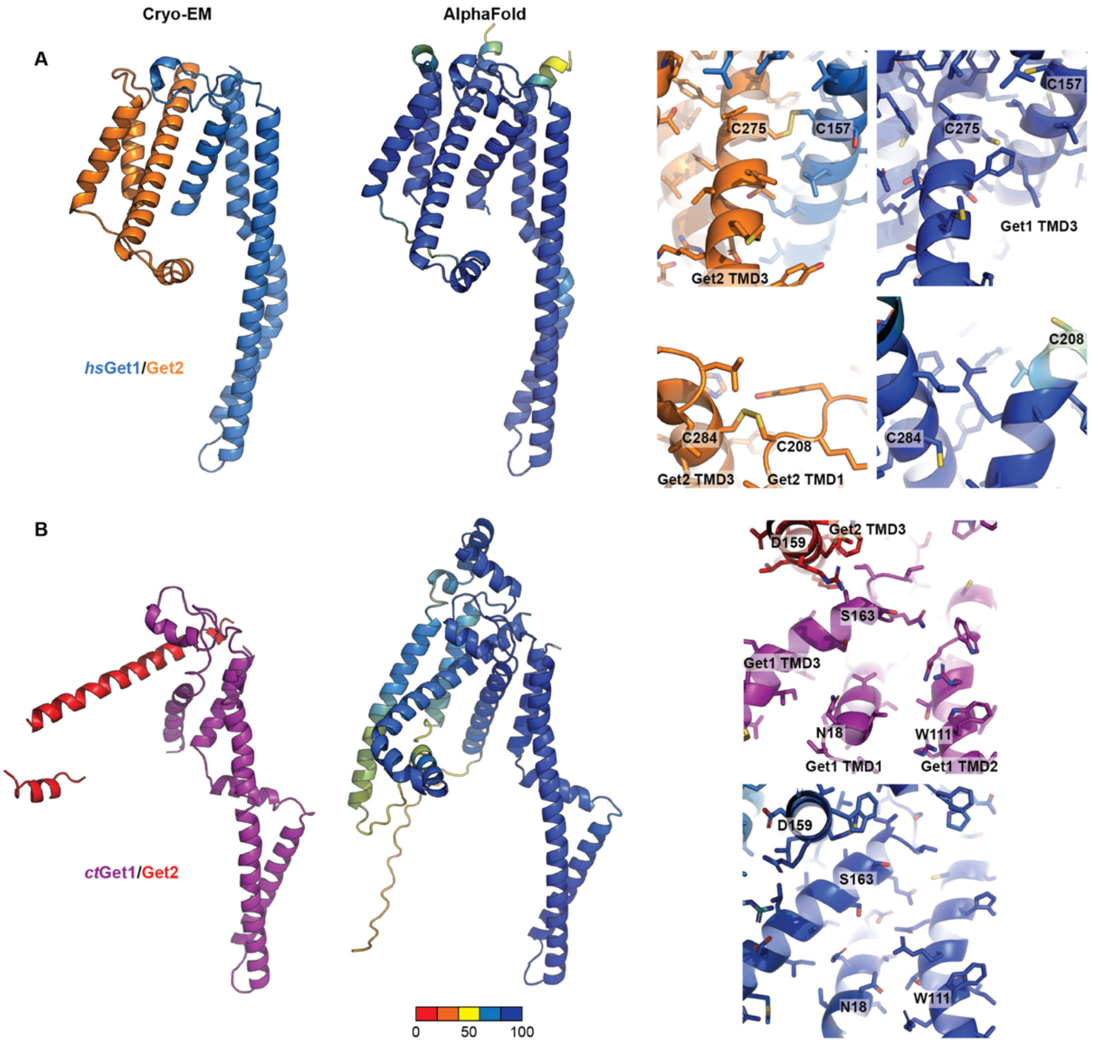

<u>**Supplementary Figure 7**</u> **– comparison of cryo-EM and AlphaFold derived models for *hs*Get1/Get2 and *ct*Get1/Get2.**

A) Side-by-side comparison of the updated *hs*Get1/Get2 heterodimer model presented in this study with an AlphaFold model (RMSD 2.37 Å over 259 Cα atoms). Clear density for disulphide bonds (Supplementary Figure 5E) results in *hs*Get1 TMD3 and *hs*Get2 TMD1 being modelled with a different register in the cryo-EM reconstruction than in the AlphaFold derived model (zoomed insets). **B)** Side-by-side comparison of the amphipol *ct*Get1/Get2 heterodimer model presented in this study with an AlphaFold model (RMSD 2.33 Å over 194 Cα atoms). The positioning and register of the TMDs are similar between both models (zoomed insets). Both AlphaFold models are the top ranked model from the predicted local distance difference test (pLDDT) after running the ColabFold AlphaFold2_advanced Jupyter notebook inside Google Colaboratory[50]. They are coloured according to pLDDT values, where pLDDT > 90 (blue) is a high estimated accuracy of backbone and side chain positions and were used to derive models for the ER cap.

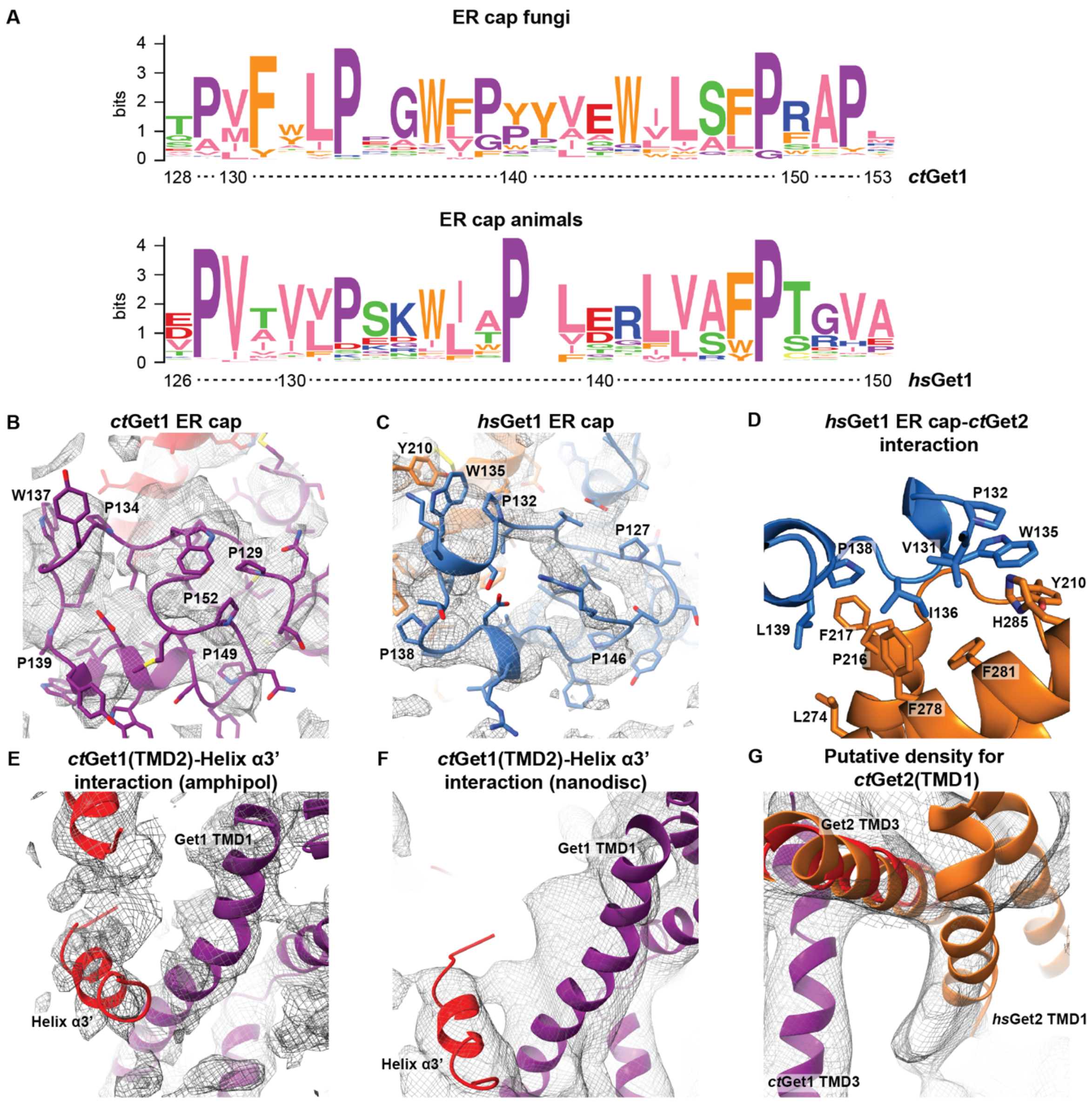

<u>Supplementary Figure 8</u> – Conserved structural features between the *ct*Get2$^{\Delta N}$-Get1/Get3 and *hs*Get2$^{\Delta N}$-Get1/Get3 complexes.

A) Sequence conservation within the ER cap of 453 fungal and 445 animal Get1 sequences. Residues in the sequence logos are numbered according to the residues in *ct*Get1 and *hs*Get1. **B-G)** Structural features within the *ct*Get2$^{\Delta N}$-Get1/Get3 and *hs*Get2$^{\Delta N}$-Get1/Get3 reconstructions. **G)** Superimposition of *ct*Get2$^{\Delta N}$-Get1 and *hs*Get2$^{\Delta N}$-Get1 as shown in Figure 2C, showing the density from the *ct*Get2$^{\Delta N}$-Get1/Get3 nanodisc reconstruction.

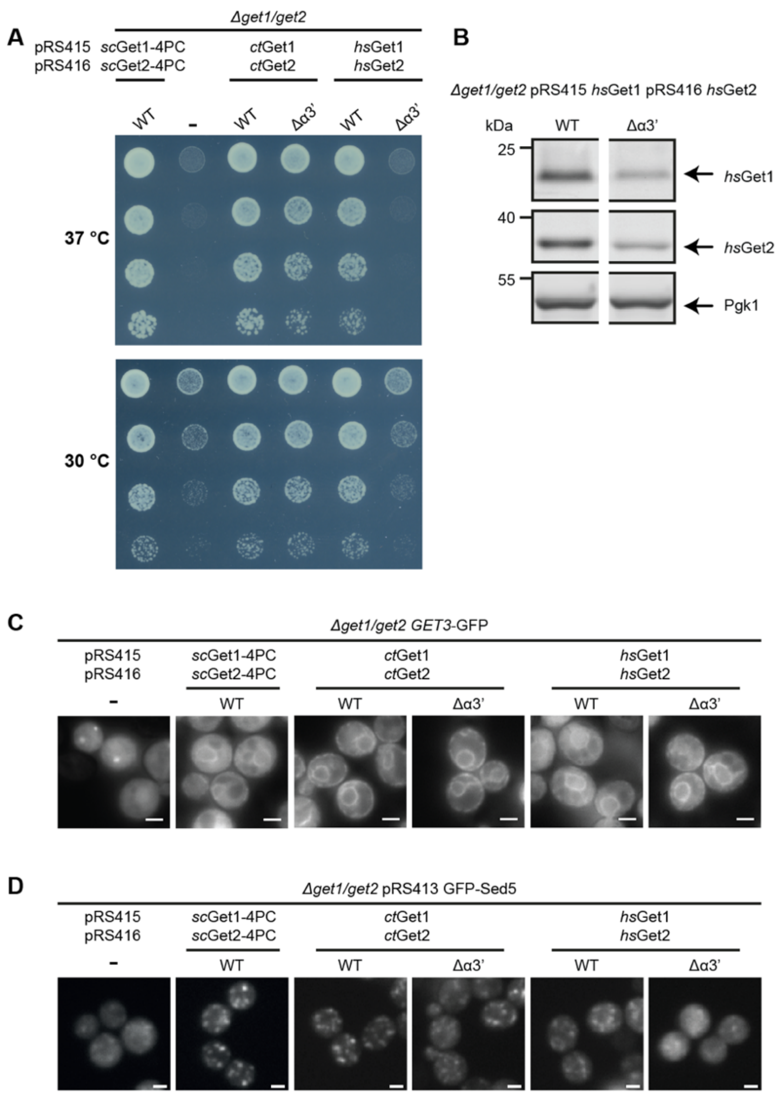

<u>Supplementary Figure 9</u> – The functional importance of Get2 helix α3'.

*sc*Get1-4PC/Get2-4PC, *ct*Get1/Get2 or *hs*Get1/Get2 were co-expressed from the indicated plasmids in *Δget1/get2* yeast strains for wild type (WT) and mutant sequences. Transformants with the empty vector were taken as a negative control (-). **A)** Serial dilutions of *Δget1/get2* yeast cells expressing *sc*Get1/Get2, *ct*Get1/Get2 or *hs*Get1/Get2 constructs spotted on synthetic dropout plates at 37°C and 30°C. The spotting assays are representative of n=3. **B)** Western blot of *hs*Get1/Get2 expression in *Δget1/get2* yeast cells. Pgk1 was analysed as a loading control. The immunoblots are representative of n=3. **C)** Representative images from fluorescence microscopy showing the subcellular localisation of *sc*Get3-GFP in *Δget1/get2* yeast cells expressing genomically tagged Get3 and *sc*Get1/Get2, *ct*Get1/Get2 or *hs*Get1/Get2 constructs. **D)** Representative fluorescence microscopy images of *Δget1/get2* cells expressing GFP-Sed5 and *sc*Get1/Get2, *ct*Get1/Get2 or *hs*Get1/Get2 constructs from separate plasmids. All scale bars represent 2 µM.

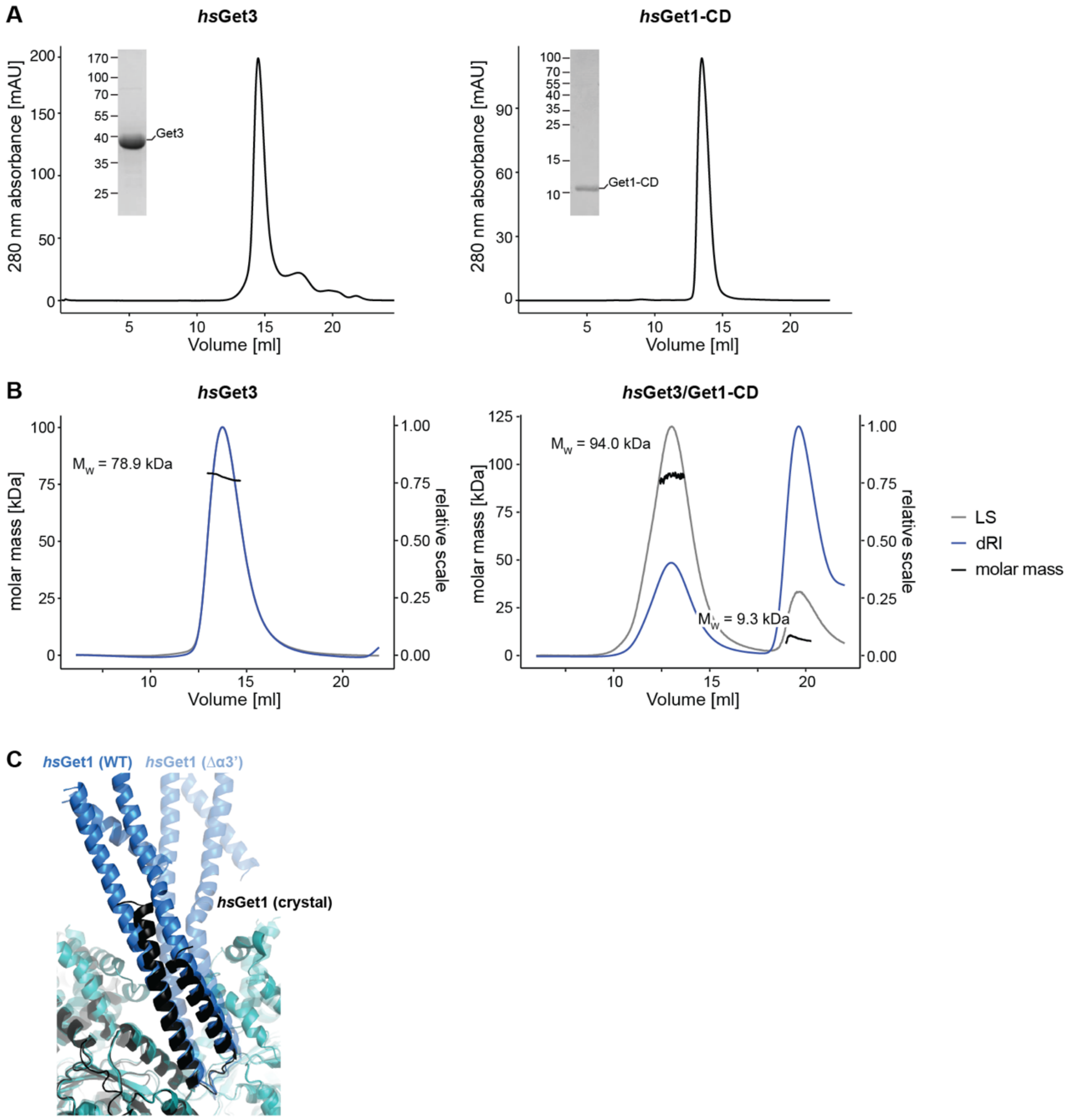

Supplementary Figure 10 – Analysis of the *hs*Get3/Get1-CD complex.

A) Size exclusion chromatography profiles for *hs*Get3 and *hs*Get1-CD. The insets show Coomassie stained SDS-PAGE gels for the final samples. **B)** Analysis of the oligomeric state of *hs*Get3 (expected 80.2 kDa for dimer) and reconstituted *hs*Get3/Get1-CD (expected 96.6 kDa for 2:2 complex, 8.2 kDa for Get1-CD alone) by size exclusion chromatography and in-line MALS. The left axis represents the molecular weight at any given point in the chromatogram. **C)** Superposition of Get3 within the *hs*Get3/Get1-CD crystal structure (black) with Get3 chain A within the WT *hs*Get2$^{\Delta N}$-Get1/Get3 (RMSD 1.48 Å over 237 Cα atoms), which is shown overlaid with *hs*Get2$^{\Delta N/\Delta\alpha3'}$-Get1/Get3 as in Figure 1C.

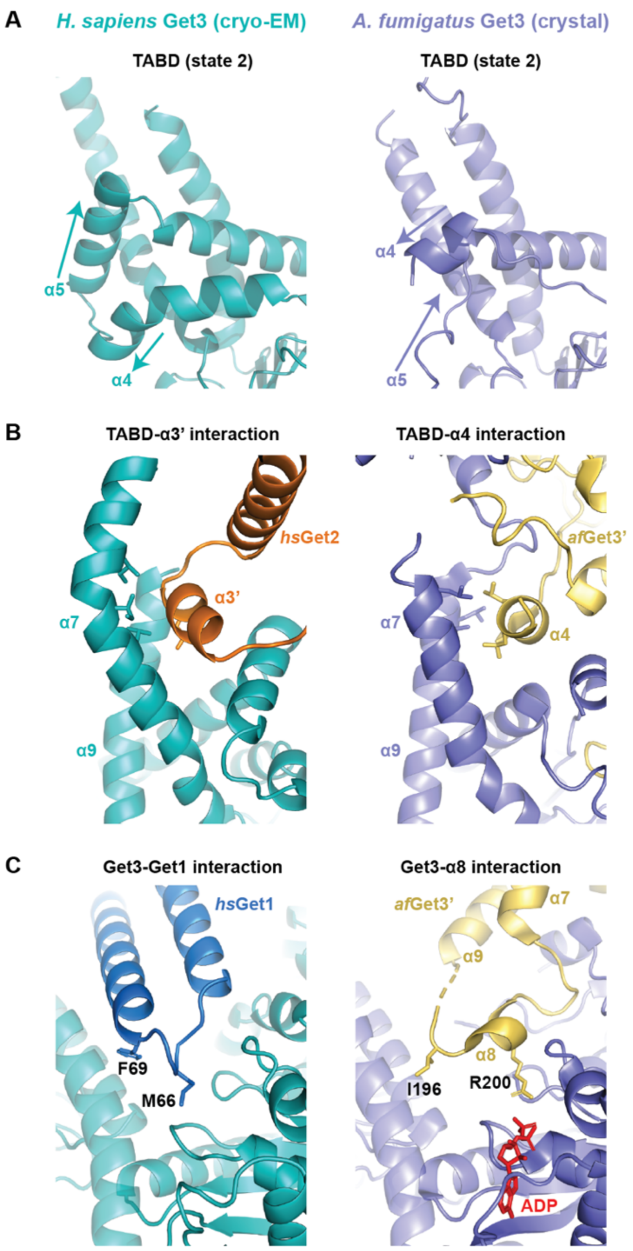

<u>Supplementary Figure 11</u> – Comparison of the wild type *hs*Get2$^{\Delta N}$-Get1/Get3 structure with the *af*Get3 crystal structure.

A) Equivalent views of the Get3 TABD after superimposition via chain A of *hs*Get3 and *af*Get3 (PDB ID 3IBG; RMSD 1.27 Å over 246 Cα atoms). The arrows show the directionality of the helices. **B)** Equivalent views of the Get3 TABD after superimposition via chain A helices α7-α9 of *hs*Get3 (residues 174-237) and *af*Get3 (residues 168-231; RMSD 2.61 Å over 262 total atoms). The interaction between *hs*Get3 and *hs*Get2 helix α3' is similar to that between *af*Get3 and *af*Get3 helix α4 from an adjacent dimer. **C)** Equivalent views of the Get3 dimer interface after superimposition as in **A**. The interaction between *hs*Get3 and the tip of the *hs*Get1-CD is similar to that between *af*Get3 and *af*Get3 helix α8 from an adjacent dimer.

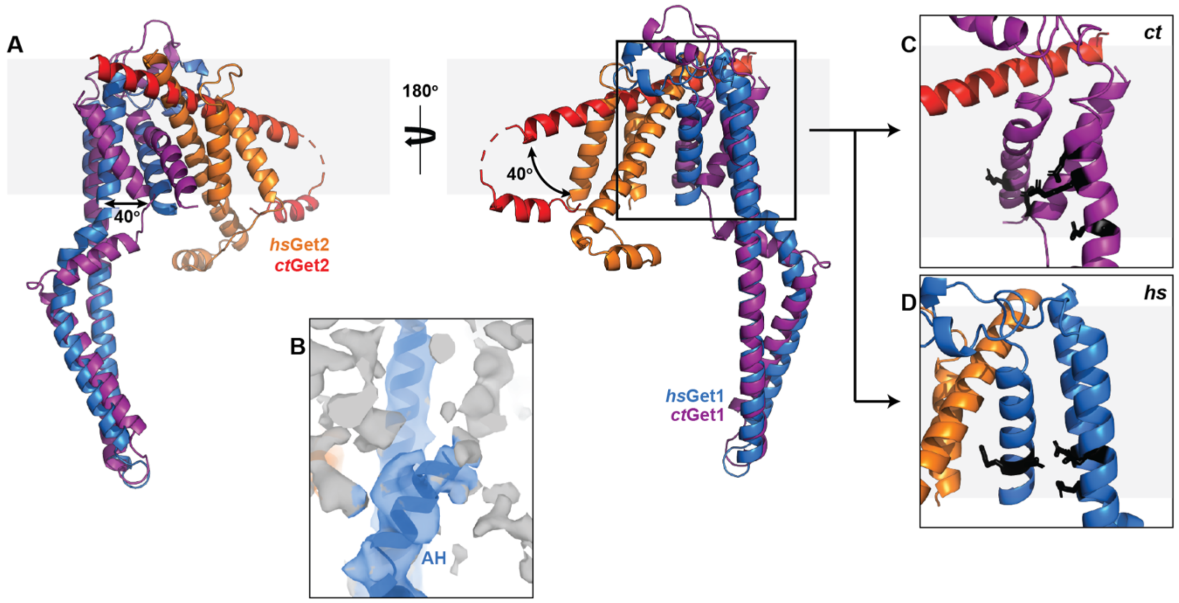

Supplementary Figure 12 – Positioning of the *ct*Get1/Get2 and *hs*Get1/Get2 heterodimers in the membrane plane.

A) Superimposition of the *hs*Get1-CD with the *ct*Get1-CD (RMSD 2.18 Å over 79 Cα atoms). The 40° change in tilt of *ct*Get1 TMD1 and *ct*Get2 TMD3 is shown. Membrane plane defined perpendicular to the Get3 symmetry axis with boundaries inferred from the location of the TMDs. **B)** Model for *hs*Get1 superimposed with the *hs*Get2$^{\Delta N/\Delta \alpha 3'}$-Get1/Get3 cryo-EM density contoured to 0.17. Within the poorly resolved *hs*Get1/Get2 heterodimer, the density beyond the AH is of insufficient quality to model *hs*Get1 TMD1 but indicates TMD1 and TMD2 are no longer arranged in parallel. **C-D)** Zoomed insets of the boxed region in **A** showing the position of the hydrophilic groove formed by **C)** *ct*Get1/Get2 TMDs and **D)** *hs*Get1/Get2 TMDs relative to the membrane plane. Hydrophilic residues pointing into the groove are shown as black sticks.

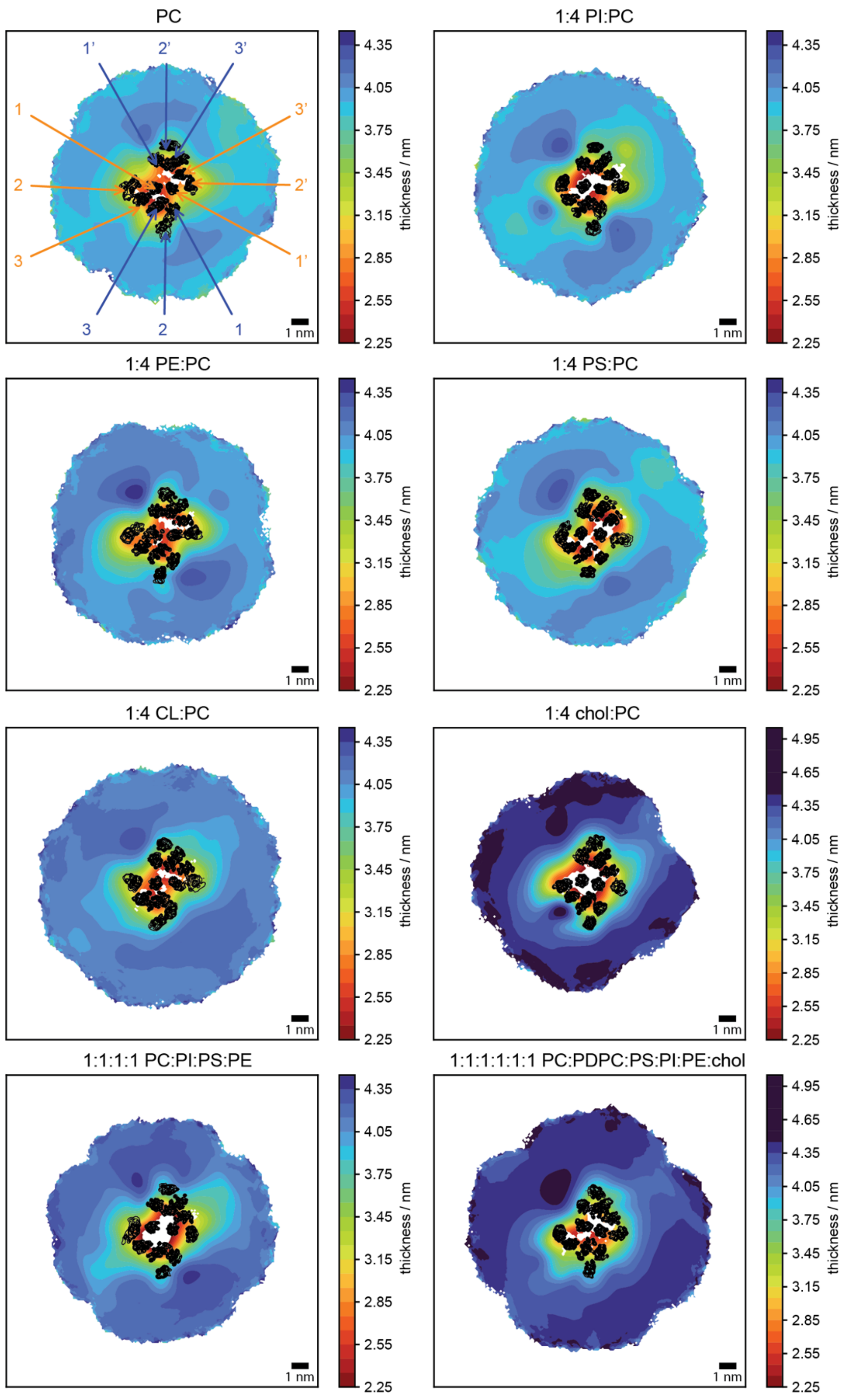

<u>Supplementary Figure 13</u> – Membrane thinning by the GET insertase is independent of bilayer composition.

Membrane thickness maps, calculated as described in the Methods section, for each lipid composition studied (Supplementary Table 2).

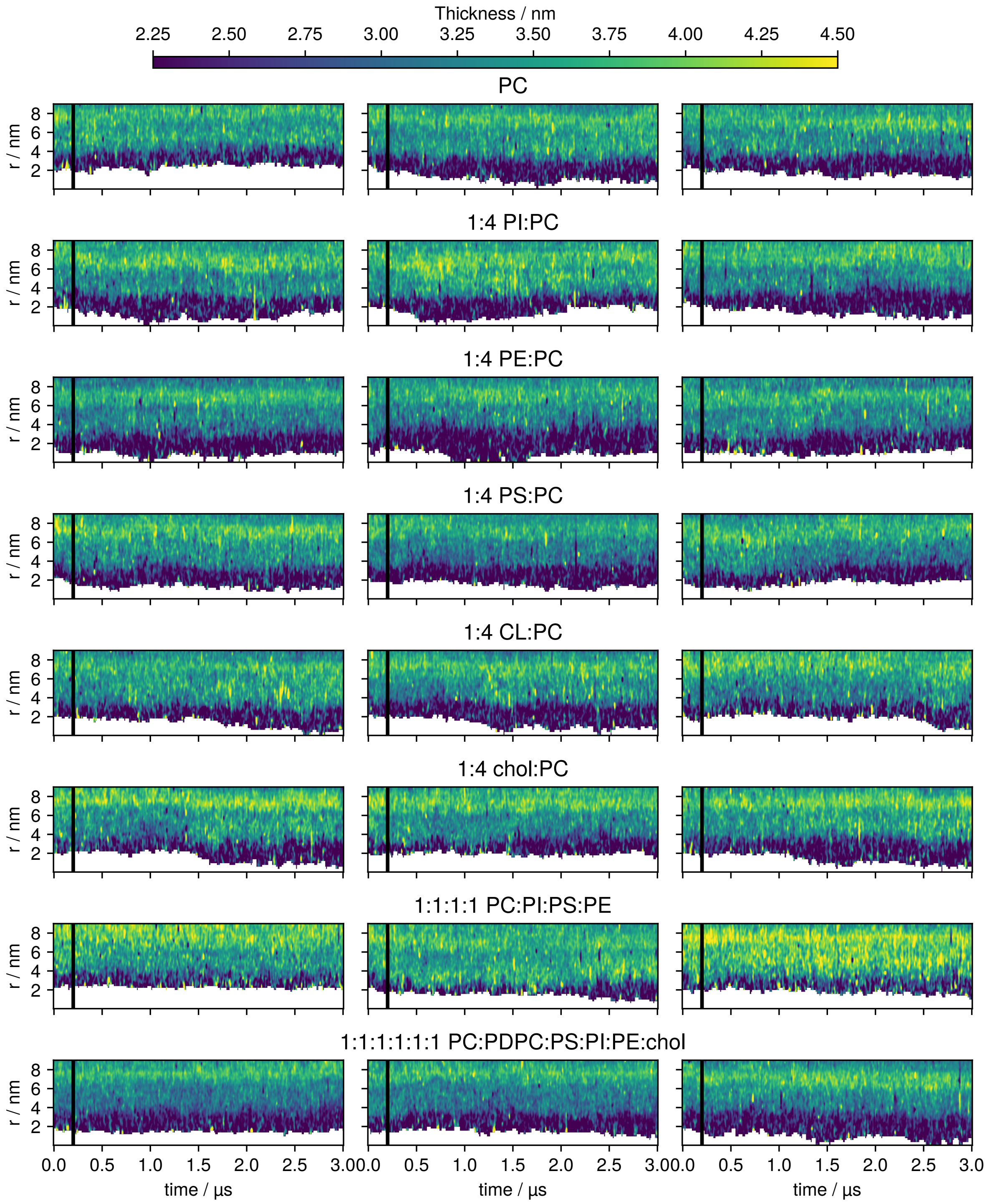

<u>Supplementary Figure 14</u> – Temporal and spatial evolution of membrane thickness. The average membrane thickness from the center of mass of the protein transmembrane domain as a function of radial lateral distance calculated for each simulation frame. Each row corresponds to the simulation system mentioned above it, and each column is an independent repeat. The vertical black lines indicate the 200 ns time point, after which the data are used for other analyses. The membrane gets thinner close to the protein, and the results are consistent throughout the simulation and across the three simulation repeats of each system.
