# Supplementary material for: The GET insertase exhibits conformational plasticity and induces membrane thinning
Source: Nat Commun. 2023 Nov 14;14:7355. doi: 10.1038/s41467-023-42867-2 (PMC10646013; doi:10.1038/s41467-023-42867-2)
Supplement: Supplementary file 3 — Description of Additional Supplementary Files [file 41467_2023_42867_MOESM3_ESM.pdf]

Description of Additional Supplementary Files

File Name: Supplementary Data 1
Description: List of all deposited structures of Get3 alone and in complex with its different binding partners used for comparison with our structures.

File Name: Supplementary Data 2
Description: List of the lipid compositions used in atomistic molecular dynamics (MD) simulations of our structure of hsGet2ΔN-Get1/Get3 embedded in a lipid bilayer of varying lipid compositions.

File Name: Supplementary Movie 1
Description: – **Membrane thinning by the human GET insertase.**
Average 3D iso-occupancy map for the lipid phosphorus atoms showing local membrane thickness and deformation relative to the structure of *hs*Get1/Get2. Both 2D membrane thickness maps and the 3D iso-occupancy maps were constructed as an average over all 3 simulation repeats after discarding the first 200 ns from each repeat, after the transmembrane region of the protein is aligned on the membrane plane.

File Name: Supplementary Movie 2
Description: **Transition from state 1 to state 2 of the heterotetramer leads to steric clashes when bound exclusively to open *hs*Get3**
A model for state 1 was constructed by superimposing the wild type *hs*Get2$^{\Delta N}$ -Get1 heterodimer on each *hs*Get1 coiled-coil in the *hs*Get2$^{\Delta N/\Delta\alpha 3'}$ -Get1/Get3 structure. The states are interconverted by using morph in ChimeraX[52] . *hs*Get3, teal; *hs*Get1, blue; *hs*Get2, orange.
