# Supplementary material for: The GET insertase exhibits conformational plasticity and induces membrane thinning
Source: Nat Commun. 2023 Nov 14;14:7355. doi: 10.1038/s41467-023-42867-2 (PMC10646013; doi:10.1038/s41467-023-42867-2)
Supplement: Supplementary file 4 — Supplementary Data 1 [file 41467_2023_42867_MOESM4_ESM.pdf]

Get3 Sequence	Binding Partner	Nucleotide	Dimer Conformation	TABD Conformation	Reference	PDB ID
Get3 in isolation						
S. cerevisiae	-	-	open	State 1 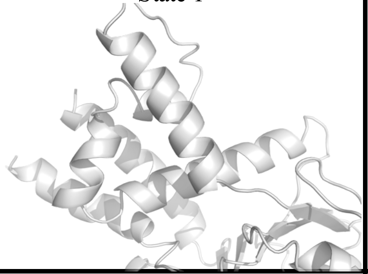	Hu *et al*., 2009	3H84
S. cerevisiae	-	-	open	State 1 (α4/α5 partially resolved) 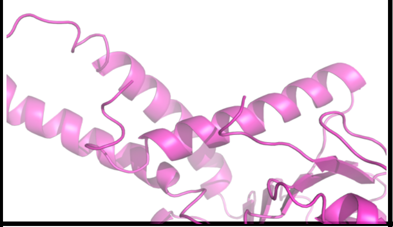	Yamagata *et al*., 2010	3A36
S. cerevisiae	-	-	monomer	Unresolved 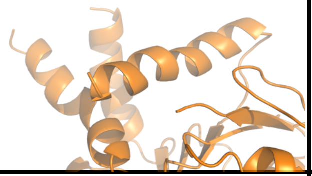	Suloway et al., 2009	3IDQ
S. pombe	-	-	open	State 1 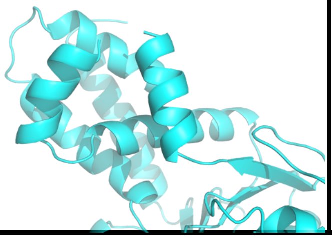	Mateja et al., 2009	2WOO

A. fumigatus	-	ADP	open	State 2 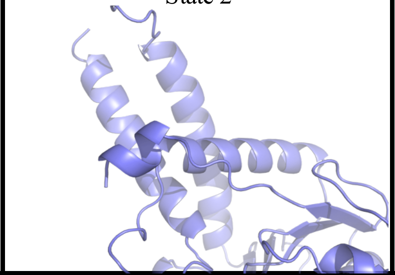	Suloway et al., 2009	3IBG
S. cerevisiae	-	ADP	open	State 1 (α4/α5 partially resolved) 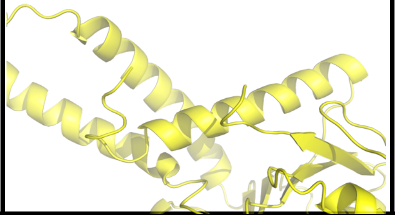	Yamagata et al., 2010	3A37
D. hansenii	-	ADP	closed	Unresolved 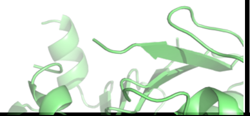	Hu et al., 2009	3IO3
C. thermophilum	-	AMPPNP-Mg^{2+}	closed	Unresolved 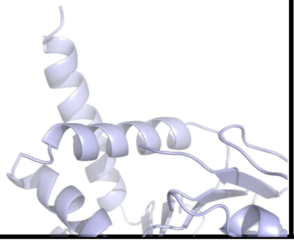	Bozkurt et al., 2009	3IQW

C. thermophilum	-	ADP-Mg^{2+}	closed	Unresolved 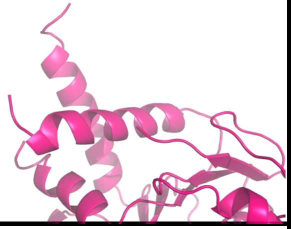	Bozkurt et al., 2009	3IQX
S. cerevisiae	-	ADP-AlF_4-Mg^{2+}	fully closed	State 1 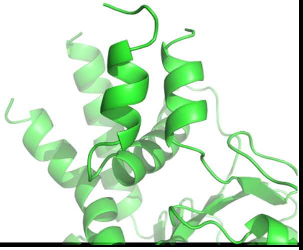	Mateja et al., 2009	2WOJ
M. thermautotrophicus	-	ADP-AlF_4-Mg^{2+}	fully closed	State 1 (α5 partially resolved) 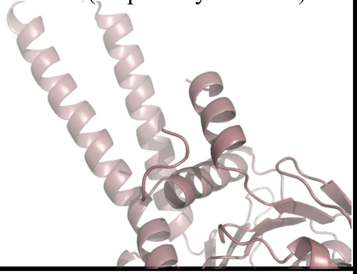	Sherrill et al., 2011	3ZQ6
M. jannaschii	-	ADP-AlF_4-Mg^{2+}	tetramer	State 1 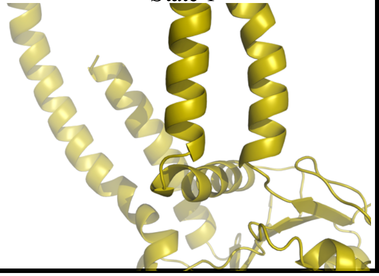	Suloway et al., 2012	3UG6

M. jannaschii	-	ADP-AlF$_4$-Mg^{2+}	tetramer	State 1 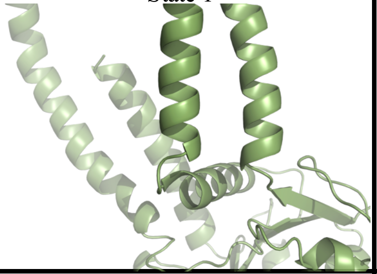	Suloway et al., 2012	3UG7
G. intestinalis (D53N)	-	ATP	closed	State 1 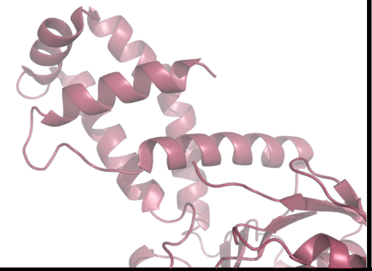	Fry et al., 2022	7SPY
G. intestinalis	-	-	open	State 1 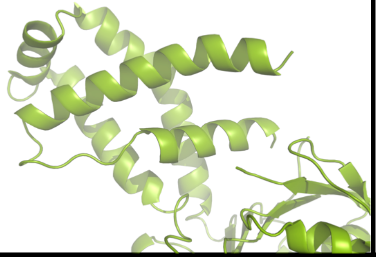	Fry et al., 2022	7SPZ

Complexes with Get1/Get2

S. cerevisiae	Get2-CD	ADP-Mg^{2+}	closed	Unresolved		3SJD
S. cerevisiae	Get2-CD	ADP-AlF$_4$-Mg^{2+}	closed	State 1 (α5 partially resolved) 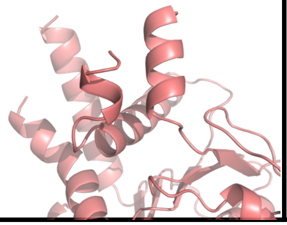	Mariappan et al., 2011	3ZS9

S. cerevisiae	Get1-CD	-	semi-open	State 1 (α4/α5 partially resolved) 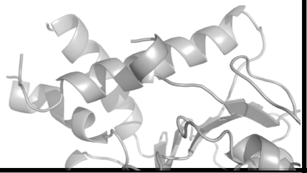	Stefer et al., 2011	3SJC
S. cerevisiae	Get1-CD	ADP	semi-open	State 1 (α4/α5 partially resolved) 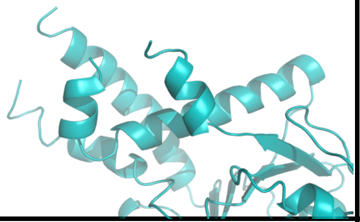	Kubota et al., 2012	3VLC
S. cerevisiae	Get1-CD	-	open	State 1 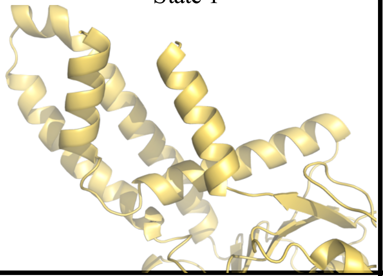	Stefer et al., 2011	3SJA
S. cerevisiae	Get1-CD	-	open	State 1 (α4/α5 partially resolved) 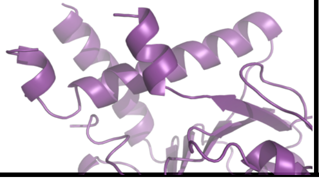	Stefer et al., 2011	3SJB

S. cerevisiae	Get1-CD	-	open	State 1 (α5 partially resolved) 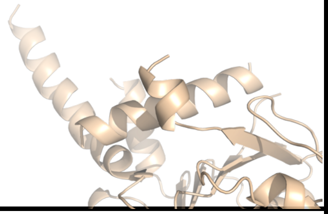	Mariappan et al., 2011	3ZS8
S. cerevisiae	Get1-CD	ADP	open	State 1 (α4/α5 partially resolved) 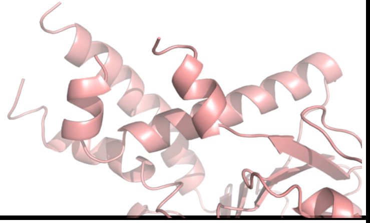	Kubota et al., 2012	3B2E
H. sapiens	WRB-CD	-	monomer	State 1 (α4 partially resolved) 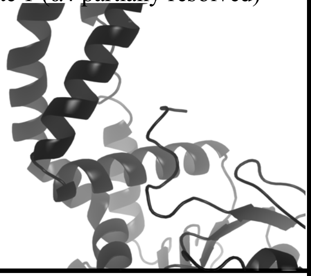	This study	8CQZ
H. sapiens	CAML$^{\Delta N}$-WRB	-	open	State 2 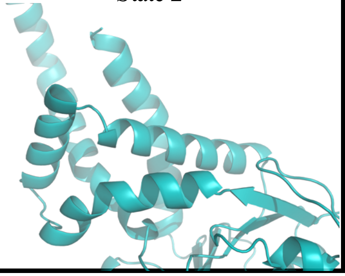	McDowell et al., 2020 and this study	6SO5 and 8CR1

H. sapiens	CAML$^{\Delta N/\Delta\alpha3'}$-WRB	-	open	Unresolved 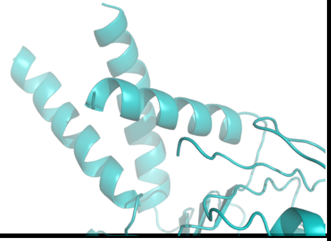	This study	8CR2
C. thermophilum	Get2$^{\Delta N}$-Get1	-	open	State 1 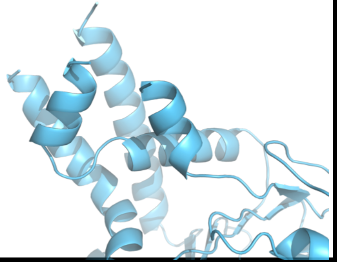	This study	8ODU
Targeting complexes						
S. cerevisiae (D57V)	Get4/Get5N	ATP	closed	State 1 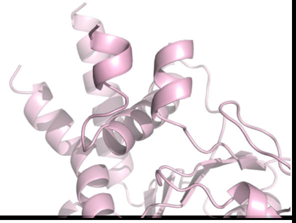	Gristick *et al*., 2014	4PWX
S. cerevisiae	Get4/Get5N	-	open	State 1 (α4/α5 partially resolved) 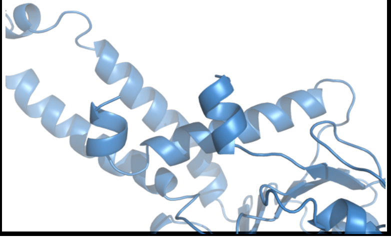	Gristick *et al*., 2015	5BW8

D. rerio (D68N)	Human cBag6/Ubl4a/ Get4/SGTA	ATP	closed	State 1 (α4 unwound) 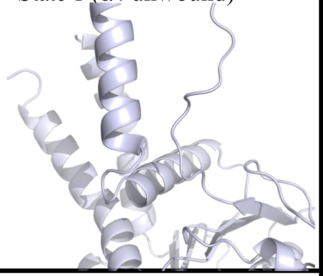	Keszei *et al*., 2021	7RUC
D. rerio (D68N)	Human cBag6/Ubl4a/ Get4	ATP	closed	State 1 (α4 unwound) 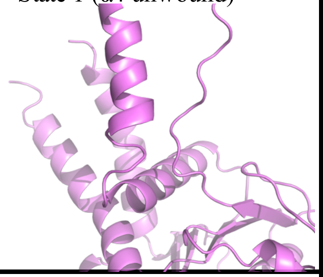	Keszei *et al*., 2021	7RUA
D. rerio (D68N)	Human cBag6/Ubl4a/ Get4	ATP	closed	State 1 (α4 unwound) 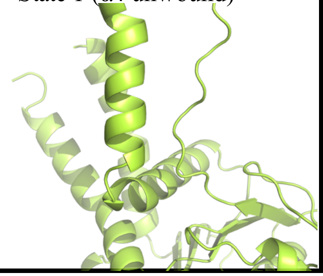	Keszei *et al*., 2021	7RU9

Substrate complexes

S. cerevisiae (D57N)	Sec22/sAB	ATP/ADP	closed	State 1 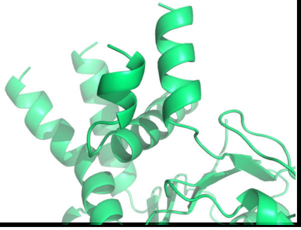	Mateja *et al*., 2015	4XWO

S. cerevisiae (D57N)	Nvy1/sAB	ATP	closed	State 1 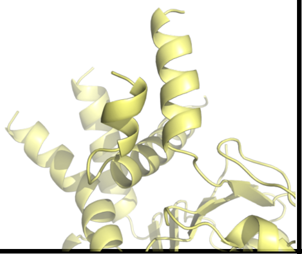	Mateja *et al*., 2015	4XVU
S. cerevisiae (D57N)	Pep12/sAB	ATP/ADP	closed	State 1 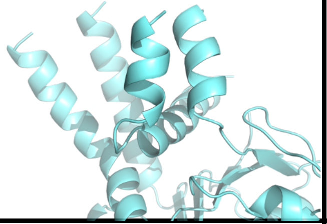	Mateja *et al*., 2015	4XTR
G. intestinalis	Bos1	ADP	post hydrolysis'+E12	State 1 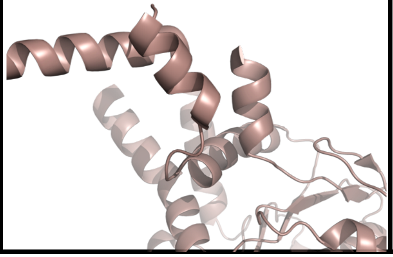	Fry et al., 2022	7SQ0
