# Supplementary material for: The GET insertase exhibits conformational plasticity and induces membrane thinning
Source: Nat Commun. 2023 Nov 14;14:7355. doi: 10.1038/s41467-023-42867-2 (PMC10646013; doi:10.1038/s41467-023-42867-2)
Supplement: Supplementary file 5 — Supplementary Data 2 [file 41467_2023_42867_MOESM5_ESM.pdf]

System No.	System Name	Membrane Composition (mol%)							System Composition (number of molecules/ions)									
		POPC	PDPC	POPS	POPI	POPE	Chol	CL	POPC	PDPC	POPS	POPI	POPE	Chol	CL	K+	Cl-	Water
1	PC	100							305							134	134	49018
2	1:4 PI:PC	80			20				244			61				193	132	48401
3	1:4 PE:PC	80				20			244				61			130	130	47765
4	1:4 PS:PC	80		20					244		61					192	131	47860
5	1:4 CL:PC	80						20	244						61	215	154	56669
6	1:4 chol:PC	80					20		260					65		131	131	48003
7	1:1:1:1 PC:PI:PS:PE	25		25	25	25			79		79	79	79			285	127	46807
8	1:1:1:1:1:1 PC:PDPC:PS:PI:PE:chol	16.7	16.7	16.7	16.7	16.7	16.7		69	69	69	69	69	69		293	155	57252
