# Supplementary material for: The GET insertase exhibits conformational plasticity and induces membrane thinning
Source: Nat Commun. 2023 Nov 14;14:7355. doi: 10.1038/s41467-023-42867-2 (PMC10646013; doi:10.1038/s41467-023-42867-2)
Supplement: Supplementary file 9 — Source data [file 41467_2023_42867_MOESM9_ESM.pdf]

Source Data

A

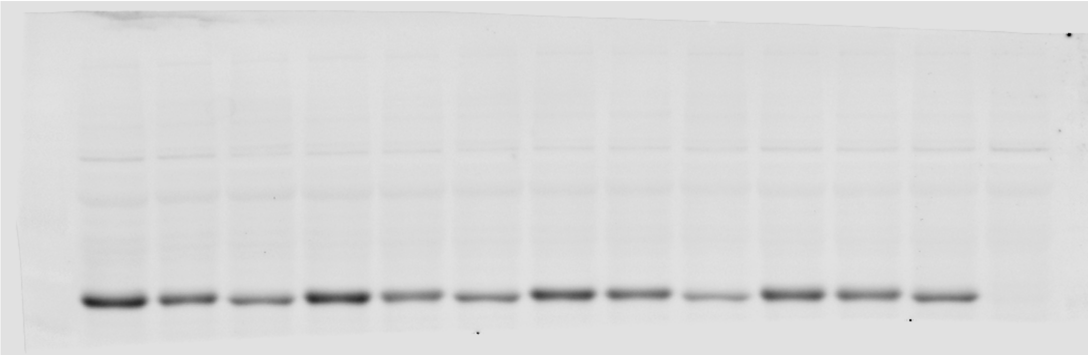

B

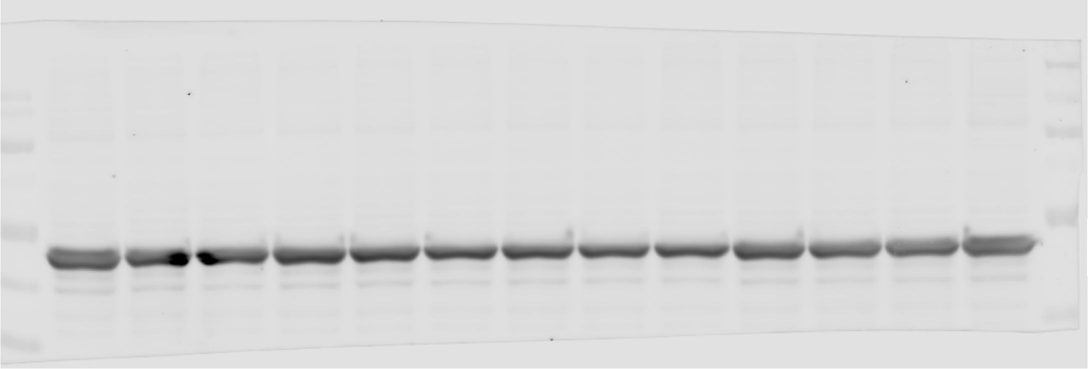

C

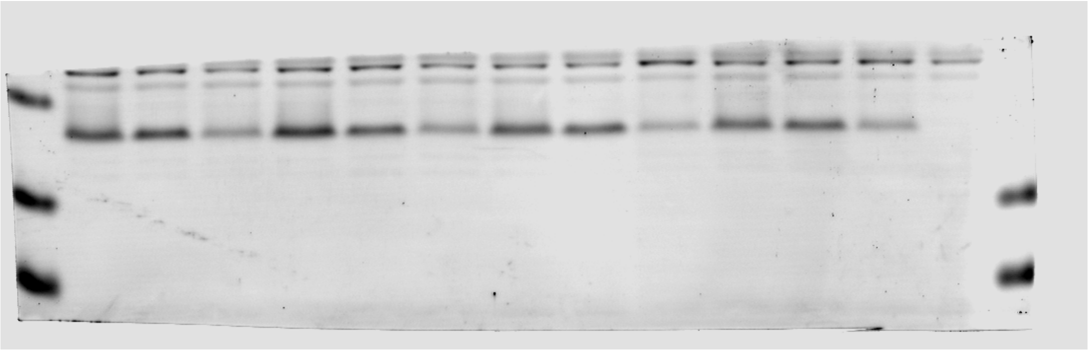

Source Data Figure 1 – Complete Western blots for Supplementary Figure 9B. The membrane was cut into two pieces horizontally, *hs*Get2 (**A**) and PGK1 (**B**) were detected on one of the pieces, and *hs*Get1 (**C**) on the other one. Loading order from left to right on all blots: protein ladder, 4x (wildtype,W158A mutant, $\Delta\alpha3'$ mutant), 1x control empty vector, protein ladder.
